# Broadband Access, Internet Use, and General Cognition in Middle-Aged and Older Adults: Longitudinal Study

**DOI:** 10.2196/75947

**Published:** 2026-03-24

**Authors:** Fangfang Cheng, Li Cao

**Affiliations:** 1 Student Affairs Office, Inner Mongolia Medical University, Hohhot, China; 2 Information Technology Department, Inner Mongolia Medical University, 5th floor, Teaching Building, Xinhua Road, Hui Min District, Hohhot, 010110, China, 86 0471-665-3023

**Keywords:** digital divide, cascade, cognitive function, trajectory, parallel-process latent growth curve modeling, PP-LGCM

## Abstract

**Background:**

Internet use may support cognitive health in aging populations, but the digital divide in access and use might worsen inequalities.

**Objective:**

This study aimed to examine whether internet use mediates the long-term relationship between broadband access and cognitive function among middle-aged and older adults in China.

**Methods:**

Using 3-wave data (2015‐2020) from the China Health and Retirement Longitudinal Study (N=6063), we applied parallel-process latent growth curve models to analyze trajectories of broadband access, internet use, and general cognition, adjusting for sociodemographic and time-varying covariates.

**Results:**

Baseline broadband access predicted initial internet use (β=0.453; *P*<.001), and broadband access increases strongly forecasted rises in internet use (β=0.625; *P*<.001). Increased internet use was associated with improved baseline general cognition (β=0.470; *P*<.001) and more significant cognitive improvements over time (β=0.444; *P*=.006). Older age, lower educational level, and rural residence were correlated with lower initial digital engagement; however, participants from rural areas demonstrated a greater adoption rate. Health status and social activities initially positively influenced outcomes; however, their impact diminished by the final wave.

**Conclusions:**

These findings demonstrate that internet use mediates the longitudinal relationship between broadband access and cognitive resilience. The observed cascading growth trajectories suggest that gradually bridging the digital divide may help mitigate cognitive decline.

## Introduction

In the digital age, the internet is essential for accessing information, maintaining social connections, and managing daily tasks [1]. Approximately 2.6 billion people worldwide (32% of the population) remain offline as of 2024 [2]. The benefits of digital innovation are unevenly distributed, leading to a persistent “digital divide” [3]. The definition of “digital divide” has shifted from a simple binary notion of connectivity in the 1990s [4] to a 3-level framework of coverage and access, use, and real-world consequences [5,6], with significant effects on individual health and societal well-being [7,8].

Cognitive decline is one of the most substantial challenges faced by the older adult population in health and social care systems [9]. Cognitive decline, which can start as early as the age of 45 years [10], is a significant risk factor for disability, dementia, and death [11-13]. A notable increase in cognitive decline after the age of 65 years has been documented [14]. China, with the world’s largest older adult population, faces an increasing challenge: almost 20% of adults aged >60 years have mild cognitive impairment, with 6% developing dementia each year [15-18].

Since 2015, China’s Internet Plus initiative has rapidly improved digital infrastructure, especially broadband access [19,20]. Despite this progress, age remains a significant factor of inequality: older adults, particularly those living in rural areas, with less education, or lacking digital skills, often face exclusion from meaningful internet use [21-23]. A 2024 cohort study in Beijing revealed that nearly half of older adults experience an “internet use divide,” which correlates with reduced processing speed and overall cognitive performance [24].

This challenge aligns with a vital global goal: the United Nations Decade of Healthy Ageing (2021‐2030) [25]. The first progress report [26] highlights that, although many countries, including China, have developed laws and infrastructure for age-friendly environments, more than two-thirds still lack sufficient resources to fully implement their action plans. This problem is especially severe in low- and middle-income areas, where nearly 80% of the world’s older adults are expected to live by 2050 [26,27]. The World Bank states that increasing access alone is insufficient to bridge the digital divide as technological progress may exclude the targeted populations [28]. Therefore, digital inclusion should be regarded as an ongoing process incorporated into age-inclusive communities and an essential component of healthy aging [29].

Empirical studies increasingly suggest that internet use—not merely access—is an active factor influencing cognitive outcomes in later stages of life. An ecological analysis found that higher broadband coverage correlates with lower prevalence of cognitive disorders, suggesting that digital infrastructure may help mitigate cognitive health disparities among older adults [30]. However, broadband access is commonly associated with an area’s economic resources, enabling internet use and social interactions. For instance, one longitudinal study in Europe found that older adults who regularly used the internet exhibited slower cognitive decline over 2 years independent of baseline connectivity [31]. Similarly, research using nationally representative data from the United States demonstrated that changes in internet use had asymmetric effects on cognitive decline in older adults: initiating use slowed decline, whereas discontinuing use accelerated it [32]. Further supporting this distinction, another investigation using data from the China Family Panel Study found that the frequency and variety of internet activities positively contributed to cognitive functions [33]. These findings align with 2 complementary theoretical perspectives. First, a widely cited model of digital inequality posits that material access to technology constitutes only the initial layer of inclusion; its benefits emerge only when individuals acquire the skills, motivation, and opportunities to engage meaningfully with digital tools [5]. Second, the cognitive reserve hypothesis proposes that participation in cognitively stimulating activities builds neural resilience against age-related cognitive decline [34]. Thus, internet use—as a dynamic, interactive, and often socially embedded behavior—may serve as the behavioral bridge through which structural access translates into sustained cognitive benefit.

**Figure 1.**
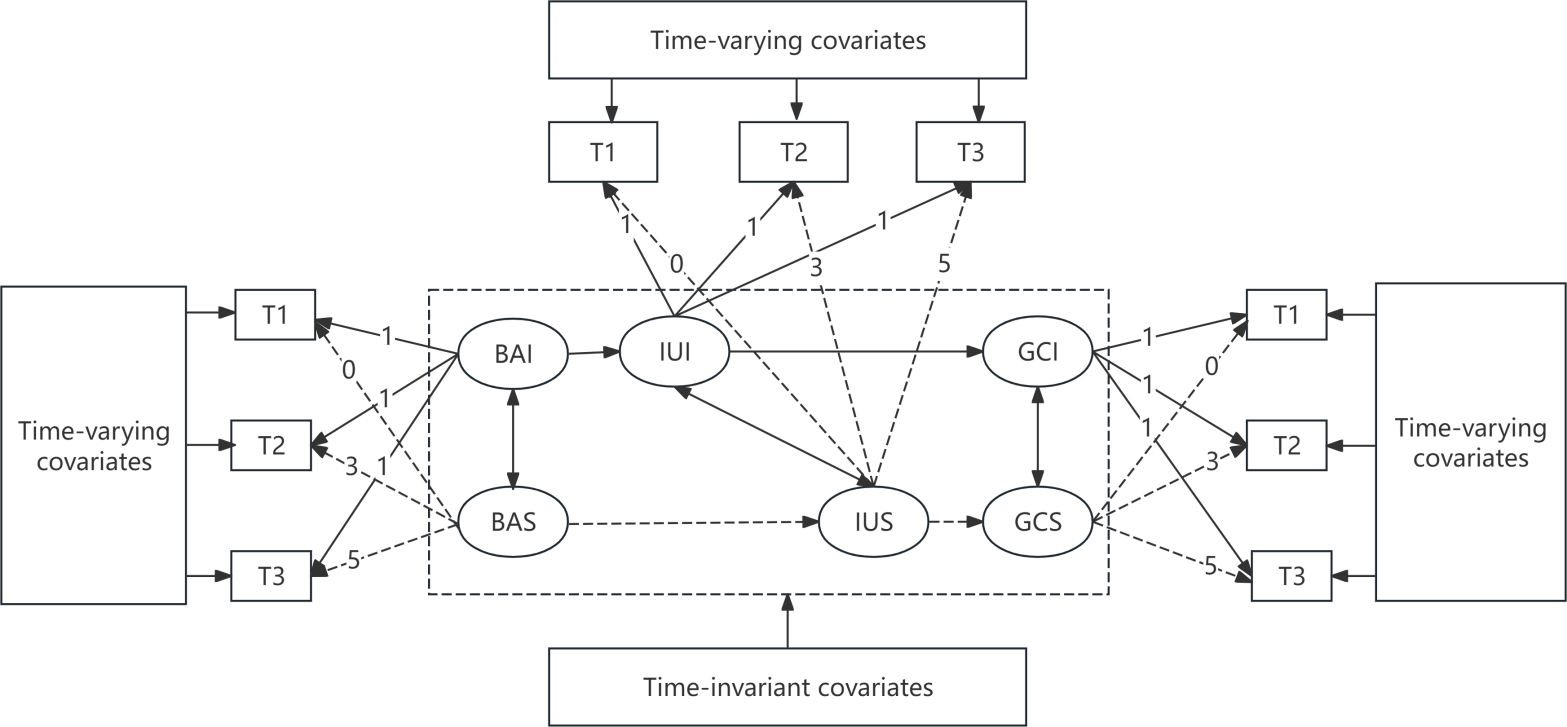
The hypothesized model of the digital divide across 3 waves. In this model, intercept factors—broadband access intercept (BAI), internet use intercept (IUI), and general cognition intercept (GCI)—capture baseline levels of broadband access, internet use, and cognitive function, respectively. Slope factors—broadband access slope (BAS), internet use slope (IUS), and general cognition slope (GCS)—represent the rates of change in these outcomes over time. T1: 2015; T2: 2018; T3: 2020.

We hypothesize that internet use mediates the longitudinal association between household broadband access and cognitive function among middle-aged and older adults in China such that growth in access predicts increased use, which in turn predicts more favorable cognitive trajectories over time as shown in Figure 1. However, most existing research examines only isolated links. These include the relationships between broadband access and cognitive outcomes, and between technology use patterns and cognitive development. Most of this research relies on cross-sectional data. Few studies use longitudinal designs capable of modeling how disparities in digital infrastructure spread through behavior to affect cognitive aging. Using China’s rapid digitization and population aging as a natural experiment, this study applied parallel-process latent growth curve modeling (PP-LGCM) with nationally representative panel data to estimate trajectories of broadband access, internet use, and general cognition simultaneously and test whether internet use changes mediate the relationship between digital infrastructure and cognitive outcomes among middle-aged and older Chinese adults.

## Methods

### Ethical Considerations

This study conducted a secondary analysis of deidentified, publicly available China Health and Retirement Longitudinal Study (CHARLS) data, exempt from additional ethics review. The original CHARLS was approved by Peking University’s Biomedical Ethics Review Committee (IRB00001052-11015), obtained written informed consent from all participants, and complied with the Declaration of Helsinki and China’s Personal Information Protection Law. All procedures adhere to CHARLS protocols.

### Participants

Participants were enrolled in 3 follow-ups of the CHARLS from 2015 to 2020. It is a nationally representative sample of the middle-aged and older adult population (aged ≥45 years) in China, a community-based longitudinal study conducted by the China Center for Economic Research at Peking University [35]. The CHARLS used a multistage, probability proportional to size sampling technique to select participants. The primary sampling units were administrative villages in rural areas and urban neighborhoods. The CHARLS data can be accessed through its official website [36]. It is designed to better understand the socioeconomic determinants and consequences of aging.

Data were drawn from 3 waves (2015 [T1], 2018 [T2], and 2020 [T3]) of the CHARLS. The baseline cohort included 23,459 participants. Of these 23,459 participants, we excluded individuals aged <45 years or >100 years and those with household consumption per capita equal to 0 at baseline (n=10,026, 42.7%), yielding 13,433 (57.3%) eligible respondents. From this group, we further excluded those with missing data on broadband access, internet use, or cognitive function (362/13,433, 2.7%) and those with a total cognitive score of 0 (143/13,433, 1.1%). An additional 1.1% (153/13,433) of the participants were excluded due to missing values for key time-invariant covariates (employment status, self-rated health, or household consumption per capita) at baseline. Of the remaining 12,775 participants with at least one valid cognitive measure, 6712 (52.5%) were lost to follow-up across the 3 waves, resulting in a final analytical sample of 6063 (47.5%) participants with sufficient longitudinal data for modeling.

### Measurements

#### Dependent Variables

Cognitive function assessment tools included an adapted Chinese version of the Mini-Mental State Examination [37]. Four specific dimensions were assessed at each wave: orientation, episodic memory, calculation ability, and constructability. Orientation was measured by naming the date (day, month, season, and year) and day of the week (score range 0-5). For episodic memory, participants were first asked to immediately repeat as many as possible of 10 Chinese nouns that were read to them and then to recall the same list 5 minutes later (score range 0-10). Calculation ability was evaluated using the serial sevens subtraction task, which required participants to subtract 7 from 100 and continue subtracting 7 from each subsequent number 5 times (score range 0-5). Constructability was evaluated through a drawing task that assesses visuospatial skills and motor coordination (score range 0-1). General cognition was the sum of cognitive scores (range 0-21) [14] and was considered the digital outcome.

#### Independent and Mediating Variables

The primary independent variable was household broadband access. The digital divide was operationalized using 2 indicators aligned with the multilevel model by van Deursen and van Dijk [5] and Scheerder et al [38], measured using the question “Does your residence have a broadband internet connection?” (yes=1; no=0). The mediating variable was internet use, measured using the question “Have you accessed the Internet in the past month?” (yes=1; no=0).

#### Covariates

There were time-invariant sociodemographic factors (age, sex, educational level, rural residence, marital status, and household consumption per capita) and time-varying characteristics (self-rated health, social activity participation, and employment status) included, all measured at each wave.

### Statistical Analysis

R (version 4.1.0; R Foundation for Statistical Computing) was used for the purposes of descriptive and correlational analyses. Descriptive statistics were used to summarize the characteristics of the participants, specifically frequencies with percentages for categorical variables and medians with IQRs for continuous variables. The *lavaan* package in R was used to perform structural equation modeling. PP-LGCM was implemented to investigate the cascading effects of digital divides on trajectories of cognitive function. For each domain, the baseline level (intercept) and the rate of change over time (slope) were modeled [39]. The cascade effect framework suggests that broadband access influences internet use, which subsequently affects the intercepts and slopes of cognitive function. Full information maximum likelihood was applied to address missing data by leveraging all available information from each participant. The following fit indexes were evaluated: the comparative fit index (CFI), Tucker-Lewis index (TLI), root mean square error of approximation (RMSEA), and standardized root mean square residual (SRMR). Conventional thresholds stipulate that CFI and TLI values above 0.90 are acceptable, with values exceeding 0.95 indicating a good fit. For RMSEA and SRMR, values below 0.05 indicate a good fit, whereas values between 0.05 and 0.08 are acceptable [40]. In the conditional latent growth curve modeling and PP-LGCM analyses, demographic variables were incorporated as covariates.

## Results

### Descriptive Statistics

**Figure 2.**
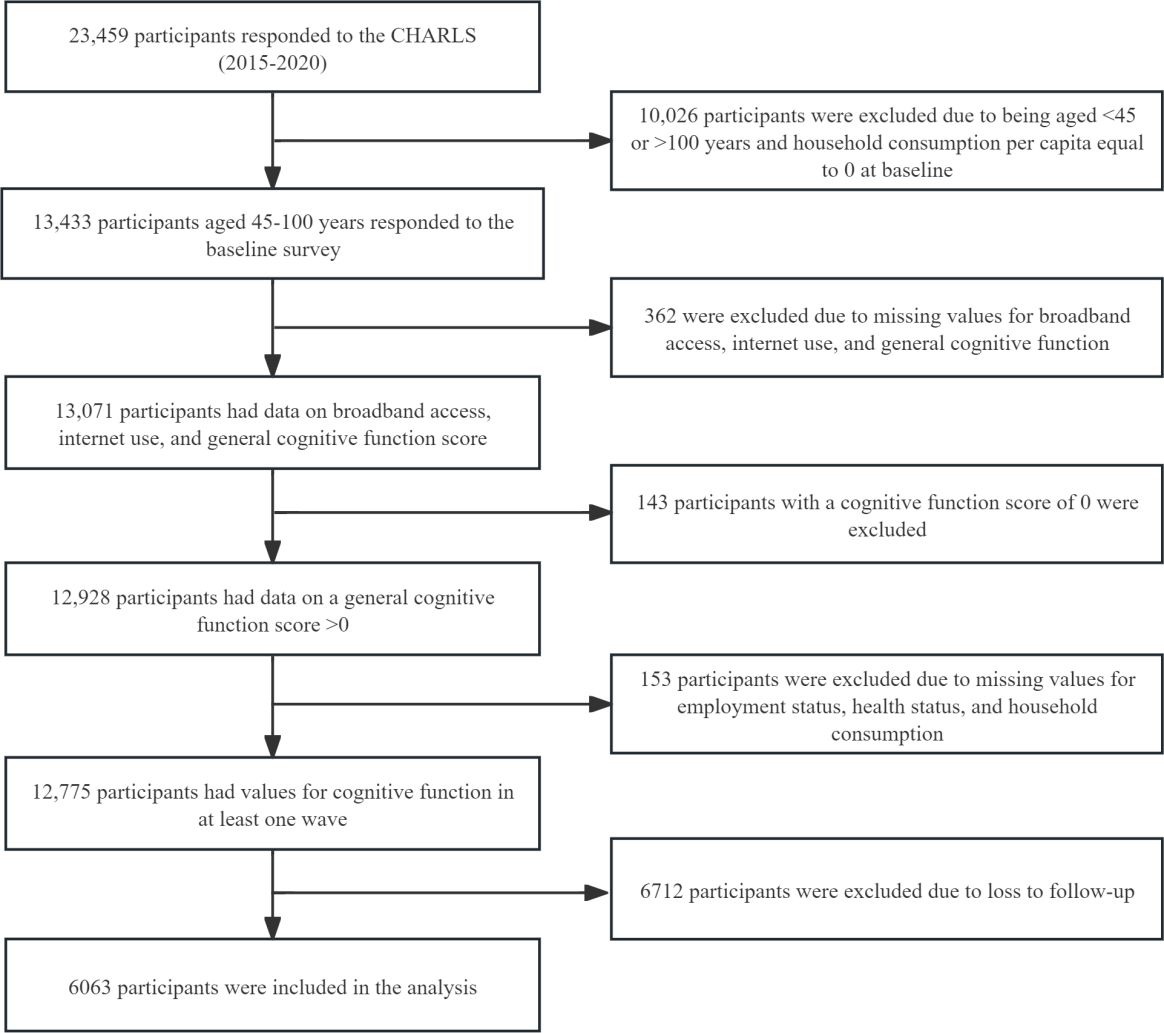
Flowchart of participant selection. CHARLS: China Health and Retirement Longitudinal Study.

A total of 6063 Chinese middle-aged and older adults were included in this study (Figure 2). Table 1 shows significant sociodemographic disparities in digital access and use. Individuals with broadband access and internet use were significantly younger, more educated, and more likely to reside in urban areas; reported better health; engaged in social activities; and had a higher household consumption (*P*<.001 in all cases). Men were more likely to be internet users (*P*<.001) but not among broadband owners (*P*=.79). Marital status and employment status showed minimal or no association with internet use (*P*=.10 and *P*=.29, respectively), although both differed slightly by broadband subscription (*P*<.001). Those who had broadband access and internet use also exhibited significantly higher cognitive function scores (median 15.5, IQR 14.5-16.5 vs median 12.5, IQR 10-14.5; *P*<.001), although the difference for broadband access was less pronounced (median 14.0, IQR 12-15.5 vs median 12.5, IQR 9.5-14.5; *P*<.001).

**Table 1.** Baseline characteristics of participants by digital divide status. The rank sum test was used for age and general cognition, and the chi-square test was used for other variables. Household consumption per capita was divided into 4 quartiles based on the 25th percentile.

Variable	No broadband access (n=4247)	Broadband access (n=1816)	*P* value	No internet use (n=5545)	Internet use (n=518)	*P* value
Age (years), median (IQR)	59 (52-65)	53 (49-59)	<.001	58 (51-64)	51 (47-56)	<.001
Male sex, n (%)			.79			<.001
No	2022 (47.6)	872 (48.0)		2708 (48.8)	186 (35.9)	
Yes	2225 (52.4)	944 (52.0)		2837 (51.2)	332 (64.1)	
Educational level, n (%)			<.001			<.001
Primary school and lower	1738 (40.9)	317 (17.5)		2048 (36.9)	7 (1.4)	
Middle school	1115 (26.3)	354 (19.5)		1419 (25.6)	50 (9.7)	
High school	964 (22.7)	642 (35.4)		1419 (25.6)	187 (36.1)	
College and higher	430 (10.1)	503 (27.7)		659 (11.9)	274 (52.9)	
Rural residence, n (%)			<.001			<.001
No	1285 (30.3)	1087 (59.9)		2000 (36.1)	372 (71.8)	
Yes	2962 (69.7)	729 (40.1)		3545 (63.9)	146 (28.2)	
Marital status, n (%)			<.001			.10
Unmarried	353 (8.3)	89 (4.9)		414 (7.5)	28 (5.4)	
Married	3894 (91.7)	1727 (95.1)		5131 (92.5)	490 (94.6)	
Health status, n (%)			<.001			<.001
Excellent	197 (4.6)	44 (2.4)		232 (4.2)	9 (1.7)	
Very good	724 (17.0)	197 (10.8)		888 (16.0)	33 (6.4)	
Good	2331 (54.9)	980 (54.0)		3048 (55.0)	263 (50.8)	
Fair	468 (11.0)	309 (17.0)		663 (12.0)	114 (22.0)	
Poor	527 (12.4)	286 (15.7)		714 (12.9)	99 (19.1)	
Social activity, n (%)			<.001			<.001
No	2142 (50.4)	668 (36.8)		2710 (48.9)	100 (19.3)	
Yes	2105 (49.6)	1148 (63.2)		2835 (51.1)	418 (80.7)	
Employment status, n (%)			<.001			.29
Not employed	1024 (24.1)	515 (28.4)		1418 (25.6)	121 (23.4)	
Employed	3223 (75.9)	1301 (71.6)		4127 (74.4)	397 (76.6)	
General cognition score (0-21), median (IQR)	12.5 (9.5-14.5)	14 (12-15.5)	<.001	12.5 (10-14.5)	15.5 (14.5-16.5)	<.001
Household consumption per capita, n (%)			<.001			<.001
Quartile 1	1267 (29.8)	250 (13.8)		1484 (26.8)	33 (6.4)	
Quartile 2	1134 (26.7)	382 (21.0)		1438 (25.9)	78 (15.1)	
Quartile 3	1022 (24.1)	493 (27.1)		1377 (24.8)	138 (26.6)	
Quartile 4	824 (19.4)	691 (38.1)		1246 (22.5)	269 (51.9)	

### Bivariate Correlations Regarding the Digital Divide

The correlation matrix presented in Table 2 delineates the relationships among broadband access, internet use, and general cognition at 3 distinct time points. The findings demonstrate moderate stability within each construct over time, with autocorrelation coefficients ranging from 0.30 to 0.62. Conversely, cross-construct correlations were consistently weaker, typically below 0.40, thereby providing evidence of adequate discriminant validity among the constructs. This pattern of associations indicates that the 3 constructs may be validly treated as separate latent variables in longitudinal modeling, such as latent growth curve analyses.

**Table 2.** Bivariate correlations (Spearman *r* and 2-tailed *P* value)^a^.

Variable	Broadband access			Internet use			General cognition		
	T1	T2	T3	T1	T2	T3	T1	T2	T3
Broadband access at T1									
*r*	1	0.45	0.34	0.35	0.31	0.33	0.21	0.22	0.23
*P* value	—^b^	<.001	<.001	<.001	<.001	<.001	<.001	<.001	<.001
Broadband access at T2									
*r*	0.45	1	0.48	0.24	0.30	0.36	0.18	0.21	0.22
*P* value	<.001	—	<.001	<.001	<.001	<.001	<.001	<.001	<.001
Broadband access at T3									
*r*	0.34	0.48	1	0.19	0.23	0.38	0.18	0.21	0.21
*P* value	<.001	<.001	—	<.001	<.001	<.001	<.001	<.001	<.001
Internet use at T1									
*r*	0.35	0.24	0.19	1	0.44	0.30	0.19	0.19	0.20
*P* value	<.001	<.001	<.001	—	<.001	<.001	<.001	<.001	<.001
Internet use at T2									
*r*	0.31	0.30	0.23	0.44	1	0.40	0.22	0.23	0.24
*P* value	<.001	<.001	<.001	<.001	—	<.001	<.001	<.001	<.001
Internet use at T3									
*r*	0.33	0.36	0.38	0.30	0.40	1	0.31	0.33	0.34
*P* value	<.001	<.001	<.001	<.001	<.001	—	<.001	<.001	<.001
General cognition at T1									
*r*	0.21	0.18	0.18	0.19	0.22	0.31	1	0.58	0.59
*P* value	<.001	<.001	<.001	<.001	<.001	<.001	—	<.001	<.001
General cognition at T2									
*r*	0.22	0.21	0.21	0.19	0.23	0.33	0.58	1	0.62
*P* value	<.001	<.001	<.001	<.001	<.001	<.001	<.001	—	<.001
General cognition at T3									
*r*	0.23	0.22	0.21	0.20	0.24	0.34	0.59	0.62	1
*P* value	<.001	<.001	<.001	<.001	<.001	<.001	<.001	<.001	—

a T1 to T3 refer to assessment time points 1 (2015), 2 (2018), and 3 (2020).

b Not applicable.

### Conditional Latent Growth Curve Models for Each Element of the Digital Divide

The fit indexes presented in Table 3 strongly support the measurement models for each domain. Overall, these results indicate that the latent construct representations were adequate over time, confirming the validity of the developmental trajectories studied.

Table 4 shows that sociodemographic factors influenced initial levels and changes over time in broadband access, internet use, and cognitive function. Older age correlated with lower baseline levels (β=–0.327 to –0.144) and slower growth in internet use (β=–0.356) and general cognition (β=–0.151). Male individuals reported slightly less broadband access (β=–0.032) but higher baseline internet use and general cognition (β=0.107), with steeper declines over time (β=–0.152 and –0.160). Higher educational level boosted initial general cognition (β=0.518) but predicted slower growth in internet use (β=–0.332) and slightly faster cognitive gains (β=0.123). Rural residents started lower (eg, broadband; β=–0.220) but showed accelerated growth in broadband access (β=0.170) and internet use (β=0.202). Health status and social activity predicted higher levels of digital inclusion early on. For example, at T2, better health status was associated with greater broadband access (β=0.066), increased internet use (β=0.081), and higher general cognition scores (β=0.039). Social activity demonstrated a stronger link specifically with internet use (β=0.122). By T3, these effects were weakened. Employment status reduced broadband access (β=–0.062 to –0.041 across waves) but had minimal impact on internet use and a small positive association with general cognition (β=0.034 at T3).

**Table 3.** Model fit indexes for conditional latent growth curve models.

Variable	Chi-square (*df*)	CFI^a^	TLI^b^	RMSEA^c^
Broadband access	93.0 (24)	0.973	0.997	0.020
Internet use	115.3 (24)	0.931	0.991	0.024
General cognition	107.6 (24)	0.991	0.983	0.025

a CFI: comparative fit index.

b TLI: Tucker-Lewis index.

c RMSEA: root mean square error of approximation.

**Table 4.** Conditional latent growth curve models (N=6063).

Covariate	Broadband access, β	Internet use, β	General cognition, β
Time-invariant covariates ~ latent intercept			
Log (age)^a^	–0.327^b^	–0.322^b^	–0.144^b^
Male sex	–0.032	0.107^b^	0.107^b^
Educational level	0.240^b^	0.457^b^	0.518^b^
Rural residence	–0.220^b^	–0.185^b^	–0.144^b^
Marital status	0.049^c^	–0.008	0.033^d^
Log (household consumption)	0.205^b^	0.213^b^	0.060^b^
Time-invariant covariates ~ latent slope			
Log (age)	–0.034	–0.356^b^	–0.151^c^
Male sex	–0.008	–0.152^c^	–0.160^b^
Educational level	–0.042	–0.332^b^	0.123^d^
Rural residence	0.170^b^	0.202^b^	0.090
Marital status	–0.018	0.027	–0.025
Log (household consumption)	–0.091^b^	–0.179^b^	–0.013
Time-varying covariates ~ observed outcomes			
Self-rated health status			
T1^e^	0.037	0.073^c^	0.046^b^
T2^f^	0.066^b^	0.081^b^	0.039^b^
T3^g^	0.016	0.006	0.028^c^
Social activity			
T1	0.034^d^	0.117^b^	0.030^b^
T2	0.074^b^	0.122^b^	0.038^b^
T3	0.020	0.109^b^	0.027^b^
Employment status			
T1	–0.062^b^	–0.003	0.007
T2	–0.073^b^	–0.014	0.013
T3	–0.041	–0.019	0.034^b^

a Log-transformed using the natural logarithm (base e).

b *P*<.001.

c *P*<.01.

d *P*<.05.

e T1: 2015.

f T2: 2018.

g T3: 2020.

### PP-LGCM Model for the Digital Divide

**Figure 3.**
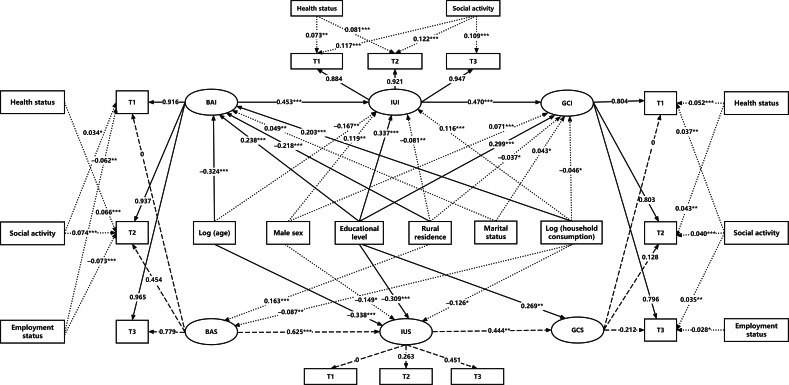
Digital divide in cognitive function using parallel-process latent growth curve modeling. For latent variables, solid lines denote intercept relationships, while dashed lines represent slope relationships. Numerical values indicate loadings from latent variables to items. For regressions of sociodemographic variables, solid lines denote statistically significant paths with |β| ≥0.20; dotted lines denote statistically significant paths with |β| <0.20; nonsignificant paths are omitted. All the coefficients are standardized. ***P<.001; **P<.01; *P<.05; BAI: broadband access intercept; BAS: broadband access slope; GCI: general cognition intercept; GCS: general cognition slope; IUI: internet use intercept; IUS: internet use slope; T1: 2015; T2: 2018; T3: 2020.

The PP-LGCM model demonstrated an excellent fit (*χ*^2^_97_=335.1; *P*<.001; CFI=0.976; TLI=0.991; RMSEA=0.020; SRMR=0.026). This finding revealed a significant mediating pathway connecting broadband access to cognitive function through internet use, as illustrated in Table 5 and Figure 3. Consistent with our hypothesized cascade model, baseline broadband access strongly predicted initial internet use (β=0.453; *P*<.001), and increases in broadband access over time forecasted steeper growth in internet use (β=0.625; *P*<.001). In turn, higher baseline internet use was associated with better initial cognition (β=0.470; *P*<.001), and greater increases in use were associated with slower cognitive decline (β=0.444; *P*=.006). Although older age, lower educational level, and rural residence were associated with lower initial digital engagement, rural participants exhibited significantly faster adoption rates (broadband slope: β=0.163; internet slope: β=0.102; *P*=.10), suggesting a partial narrowing of the use gap. Educational level boosted broadband access (β=0.238), internet use (β=0.337), and general cognition (β=0.299; *P*<.001 in all cases); and older age negatively affected broadband access (β=–0.324) and internet use (β=–0.167; *P*<.001 in both cases) and slowed internet use growth (β=–0.338; *P*<.001), with no significant impact on cognitive slope. Men reported higher baseline internet use (β=0.119; *P*<.001) and general cognition (β=0.071; *P*<.001), although they experienced steeper declines in internet use over time (β=–0.149; *P*=.02). Household consumption predicted higher baseline broadband access (β=0.203) and internet use (β=0.116; *P*<.001 in both cases) but was negatively related to their slopes. Social activity had a consistent association with internet use, with β coefficients of 0.117 at T1, 0.122 at T2, and 0.109 at T3. Furthermore, it positively predicted cognitive function (β=0.037 at T1, β=0.040 at T2, and β=0.035 at T3). Health status demonstrated a positive relationship with both internet use and cognition at T1 and T2; however, this association did not persist at T3. Employment displayed a negative correlation with broadband access across all waves (β=–0.062 at T1, β=–0.073 at T2, and β=–0.041 at T3), indicating lower adoption rates among employed individuals. Its influence on cognition remained negligible.

**Table 5.** Parallel-process latent growth curve model (N=6063).

Outcome and predictor	β (SE; 95% CI)	*P* value
Internet use intercept		<.001
Broadband access intercept	0.453 (0.022; 0.410 to 0.496)	
Internet use slope		<.001
Broadband access slope	0.625 (0.067; 0.494 to 0.756)	
General cognition intercept		<.001
Internet use intercept	0.470 (0.027; 0.417 to 0.523)	
General cognition slope		.006
Internet use slope	0.444 (0.161; 0.128 to 0.760)	.006
Broadband access intercept		
Log (age)^a^	–0.324 (0.020; –0.363 to –0.285)	<.001
Male sex	–0.032 (0.017; –0.065 to 0.001)	.07
Educational level	0.238 (0.018; 0.203 to 0.273)	<.001
Rural residence	–0.218 (0.018; –0.253 to 0.183)	<.001
Marital status	0.049 (0.018; 0.014 to 0.084)	.008
Log (household consumption)	0.203 (0.017; 0.170 to 0.236)	<.001
Broadband access slope		
Log (age)	–0.033 (0.030; –0.092 to 0.026)	.27
Male sex	–0.008 (0.028; –0.063 to 0.047)	.78
Educational level	–0.040 (0.028; –0.095 to 0.015)	.15
Rural residence	0.163 (0.028; 0.108 to 0.218)	<.001
Marital status	–0.018 (0.028; –0.073 to 0.037)	.51
Log (household consumption)	–0.087 (0.026; –0.138 to –0.036)	.001
Internet use intercept		
Log (age)	–0.167 (0.032; –0.229 to –0.105)	<.001
Male sex	0.119 (0.025; 0.070 to 0.168)	<.001
Educational level	0.337 (0.029; 0.280 to 0.394)	<.001
Rural residence	–0.081 (0.026; –0.132 to –0.030)	.001
Marital status	–0.030 (0.028; –0.085 to 0.025)	.28
Log (household consumption)	0.116 (0.025; 0.067 to 0.165)	<.001
Internet use slope		
Log (age)	–0.338 (0.073; –0.481 to –0.195)	<.001
Male sex	–0.149 (0.061; –0.269 to –0.029)	.02
Educational level	–0.309 (0.070; –0.446 to –0.172)	<.001
Rural residence	0.102 (0.062; –0.019 to 0.223)	.10
Marital status	0.038 (0.064; –0.087 to 0.163)	.55
Log (household consumption)	–0.126 (0.061; –0.246 to –0.006)	.04
General cognition intercept		
Log (age)	–0.021 (0.022; –0.064 to 0.022)	.33
Male sex	0.071 (0.018; 0.036 to 0.106)	<.001
Educational level	0.299 (0.023; 0.254 to 0.344)	<.001
Rural residence	–0.037 (0.019; –0.074 to 0.000)	.046
Marital status	0.043 (0.018; 0.008 to 0.078)	.01
Log (household consumption)	–0.046 (0.018; –0.081 to –0.011)	.01
General cognition slope		
Log (age)	0.006 (0.094; –0.178 to 0.190)	.95
Male sex	–0.091 (0.066; –0.220 to 0.038)	.17
Educational level	0.269 (0.087; 0.098 to 0.440)	.002
Rural residence	0.003 (0.061; –0.117 to 0.123)	.96
Marital status	–0.040 (0.057; –0.152 to 0.072)	.48
Log (household consumption)	0.062 (0.066; –0.067 to 0.191)	.35
Broadband access at T1^b^		
Health status	0.037 (0.019; 0.000 to 0.074)	.06
Social activity	0.034 (0.017; 0.001 to 0.067)	.049
Employment status	–0.062 (0.019; –0.099 to –0.025)	.001
Broadband access at T2^c^		
Health status	0.066 (0.019; 0.029 to 0.103)	<.001
Social activity	0.074 (0.016; 0.043 to 0.105)	<.001
Employment status	–0.073 (0.020; –0.112 to –0.034)	<.001
Broadband access at T3^d^		
Health status	0.016 (0.019; –0.021 to 0.053)	.40
Social activity	0.020 (0.019; –0.017 to 0.057)	.23
Employment status	–0.041 (0.021; –0.082 to 0.000)	.05
Internet use at T1		
Health status	0.073 (0.026; 0.022 to 0.124)	.006
Social activity	0.117 (0.024; 0.070 to 0.164)	<.001
Employment status	–0.003 (0.037; –0.076 to 0.070)	.93
Internet use at T2		
Health status	0.081 (0.023; 0.036 to 0.126)	<.001
Social activity	0.122 (0.019; 0.085 to 0.159)	<.001
Employment status	–0.014 (0.028; –0.069 to 0.041)	.56
Internet use at T3		
Health status	0.006 (0.019; –0.031 to 0.043)	.75
Social activity	0.109 (0.015; 0.080 to 0.138)	<.001
Employment status	–0.019 (0.022; –0.062 to 0.024)	.33
General cognition at T1		
Health status	0.052 (0.013; 0.027 to 0.077)	<.001
Social activity	0.037 (0.012; 0.013 to 0.061)	.002
Employment status	0.006 (0.015; –0.023 to 0.035)	.67
General cognition at T2		
Health status	0.043 (0.013; 0.018 to 0.068)	.001
Social activity	0.040 (0.012; 0.016 to 0.064)	<.001
Employment status	0.006 (0.017; –0.027 to 0.039)	.67
General cognition at T3		
Health status	0.023 (0.013; –0.002 to 0.048)	.07
Social activity	0.035 (0.012; 0.011 to 0.059)	.003
Employment status	0.028 (0.015; –0.001 to 0.057)	.046

a Log-transformed using the natural logarithm (base e).

b T1: 2015.

c T2: 2018.

d T3: 2020.

## Discussion

### Principal Results

The findings of this study demonstrated that the cascading potential influence of household broadband access predicts internet use and subsequent codevelopment of this prediction, both of which are associated with a possible slower decline in cognitive trajectories among middle-aged and older Chinese adults. The PP-LGCM model confirmed the 3-level digital divide framework and extends the perspective from static views to a process-based approach, emphasizing how disparities in infrastructure could influence actual internet use and cognitive aging through codevelopmental pathways. Our findings substantially enhance the discussion of digital inclusion as a vital component of healthy aging, emphasizing the importance of not only physical access but also creating supportive environments that enable older adults to thrive.

This aligns with the first progress report of the United Nations Decade of Healthy Ageing, which underscores the vital role of digital engagement in sustaining intrinsic capacity in later life [26]. The report cautions that, despite investments in connectivity, the resources for engagement remain insufficient, especially among older adults in low- and middle-income countries. Van Dijk [41] similarly argues that access alone is insufficient; individuals require motivation, digital skills, and supportive opportunities to translate connectivity into benefit. Our results support this view: China’s infrastructure expansion from 2015 to 2020 narrowed the rural-urban gap in broadband access, yet this did not translate into comparable gains in internet use or cognitive outcomes among rural respondents. Early advantages among urban and highly educated individuals likely reflect the Matthew effect [42,43], in which initial resources accumulate over time. However, what appears to be a cumulative advantage may stem from persistent conversion gaps [44,45]: the failure to translate access into meaningful use, especially in mid to late life, when cognitive resilience is more vulnerable [46,47].

Moreover, the association between internet use and cognitive resilience is plausibly mediated by neurocognitive mechanisms aligned with the cognitive reserve hypothesis [48,49]. Internet use is positively associated with cognitive stimulation and cognitive reserve [50,51]. Activities such as seeking health information, comparing services, or navigating digital interfaces engage executive functions, working memory, and attention [52,53]. Social relationship factors are correlated with cognitive function and its decline, and engaging in social activities via calls or messaging may reduce social isolation, which is identified as a risk factor for cognitive decline [54,55]. The internet offers problem-solving opportunities through online tasks such as navigating websites, managing banking, or learning, which demand cognitive effort and decision-making, helping maintain mental agility [56] and thereby stimulating cognitive abilities [57]. Although our binary measure of internet use limits granularity, the findings are consistent with those of research linking online behaviors to delayed recall and processing speed [58], indicating that continuous engagement activates these neural pathways [59].

Structural inequalities shape both digital and cognitive trajectories [60]. Age is a predictor of disadvantage [23,43]: older adults tend to have less broadband access, lower internet use, and slower progress in these domains. Educational level functions as a lifelong enabler, increasing broadband access, internet use, and cognitive capacity [60]. Men in this study reported higher initial levels of internet use and general cognition. However, there is a significant decline in internet use over time, rather than in cognitive function, aligning with previous research [18,61], some of which is potentially attributable to cohort attrition in men. Household consumption predicted higher initial digital engagement but also correlated with slower growth, implying saturation among more advantaged individuals [22]. Self-rated health and social activity were associated with better outcomes in the initial wave but lost significance in later waves, potentially due to survivor bias or the stabilization of digital habits [32]. Employment status consistently showed a negative relationship with broadband access across waves, likely reflecting work-related constraints or measurement limitations.

While our PP-LGCM model supports a directional cascade from access to use to general cognition, we acknowledge the plausibility of reverse causation: individuals with higher baseline cognitive capacity may be more likely to adopt and sustain internet use [32]. Future studies using cross-lagged panel models or instrumental variables are recommended to further elucidate the causal direction.

### Limitations

This study has several limitations. First, attrition (6712/12,775, 52.5%) may introduce bias if those lost to follow-up were disproportionately cognitively impaired or digitally excluded. Second, our binary measures of internet use could not distinguish between passive (eg, scrolling) and cognitively stimulating activities (eg, learning and problem-solving)—a key nuance for future research. Third, although the Mini-Mental State Examination cognitive composite is widely used in surveys among large aging populations, it has limited ceiling effects in high-functioning individuals, sensitivity to early or specific decline, and possible cultural or educational biases in literacy or numeracy items [62,63].

### Conclusions

This study provides longitudinal evidence that internet use mediates the association between broadband access and general cognition among middle-aged and older Chinese adults. The growth trajectory in broadband access predicted increased internet use, which in turn predicted slower cognitive decline. These results argue for integrating digital literacy and socially engaging online opportunities into public health strategies for healthy aging.

## Abbreviations

CFI: comparative fit index
CHARLS: China Health and Retirement Longitudinal Study
PP-LGCM: parallel-process latent growth curve modeling
RMSEA: root mean square error of approximation
SRMR: standardized root mean square residual
TLI: Tucker-Lewis index
